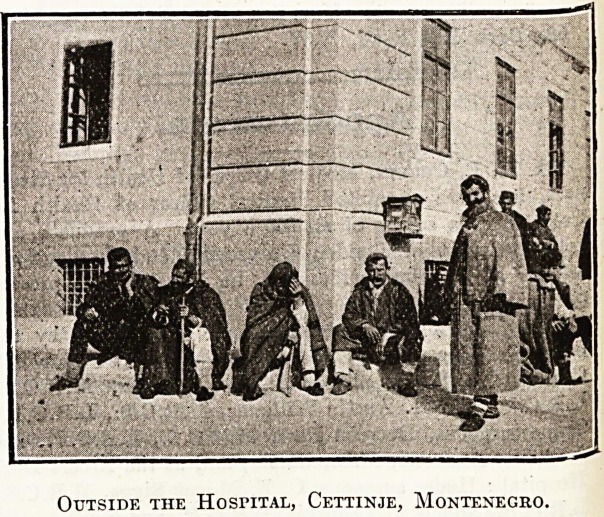# With the Wounded in Bulgaria

**Published:** 1913-11-08

**Authors:** 


### BRITISH WORKERS in FOREIGN COUNTRIES.

### With the Wounded in Bulgaria.

On November 8, 1912, a friend and I left London to nurse in the Balkans, with a letter of introduction to H.M. Queen Eleonora of Bulgaria. It was an exciting moment, for we had little idea of what the future held in store for us. Our destination was Sofia, Bulgaria, where we had heard before starting there was a large base hospital for 2,000 wounded. The journey out was full of interest, especially the latter part, as we neared the war zone.

We reached Belgrade at eleven o'clock one night, and gathered from the excited throng at the station that there was no train till next morning at 6 A.M. This was an unexpected shock, for we did not know where to go, and of course could not understand a word of what was going on around us. Fortunately a man who knew a little German came to our rescue, and told us the name of an hotel. So we got into a dilapidated species of carriage and off we started.

Group of In-patients: Montenegrins.

### A Midnight Drive.

Never shall I forget that drive! It was pitchy dark, and there had been a heavy fall of snow, which added to the usual bad state of the roads. We were jolted and jerked and plunged in and out of pitfalls. The only thing to do was to cling on tight! Of course, as luck would have it, the hotel was crammed, so off we plunged again into the dark night, and at last arrived at what we should consider in England a very fourth-rate "pub."! Here there were only a few men drinking and playing cards; they looked rather startled at the sudden appearance of two English nurses at 1 A.M. in the morning. Saved again by one who could stammer a few words of bad French, we were taken upstairs to an enormous barrack-like room as cold as a vault—at least it felt like that—a damp, penetrating cold.

Bed was out of the question; besides, we had already begun to eye with apprehension anything in the nature of bedclothes. The thing was to unfreeze, so we just got into all the coats we possessed and curled up for a few hours' sleep. Five A.M. came far too soon, with a crashing at the door, and off we had to go. Our train left at 7 o'clock, and on we crawled all day, hoping to get to Sofia some time during the afternoon, but, owing to the heavy fall of snow, we gathered we should not arrive till next day. That night was rather different from the preceding one, and we were wedged up tight in a very stuffy carriage between Bulgarians. They were really most kind to us, and occasionally even the window was opened a crack out of consideration for our feelings. They seemed very impressed that two English "sisters" should be so far from home. The little stations *en route* did indeed look warlike; masses of soldiers and rough-looking men all shouting, pushing, and jostling. At 6 A.M. we got to Sofia.

### Queen Eleonora and the Hospital.

After a few days' wait, during which time we were shown round some of the hospitals by an English lady and got a rough idea of our surroundings, we were presented to her Majesty, who asked us to help the Austrian Mission at the Ecole Militaire, an enormous building which had been converted into a temporary hospital. The building was divided into three floors; the Austrians had the middle one. We were each asked to take charge of a long room with partitions, containing roughly between 150 and 200 patients.

We started work next day, and at first it did seem a hopeless task. There were no trained nurses besides ourselves, but any number of Bulgarian ladies had come forward to help. These were called "Samaritaines." A few had had first-aid courses, but the majority no training at all, and so the nursing of the patients was practically nil; but, considering all things, one could not fail to admire the plucky start they had made. Many were anxious and keen to learn, and were only too pleased to be shown the right way to do things. Their touch was so extraordinarily gentle; it often struck one what excellent nurses they would make if trained. Never have I had more delightful patients! Their patience and pluck were beyond words, and you never heard a grumble; so grateful, too, for all that was done.

Outside the Hospital, Cettinje, Montenegro.

The language was the great difficulty, and though the ladies were very good about translating for us (many of them could speak French or German), still it was a great drawback, and one often felt the patients suffered unnecessarily. Happily they soon got to know how much we wanted to help them, and all day long there was an incessant cry for the "Sestro Engleska" (English sister). Also we soon picked up some words and could make ourselves understood a bit. I longed to talk to them and to hear some of their stories, which must have been most interesting. A few wrote down their experiences for me, which were translated into English by a Bulgarian girl. The following is one of them:—

### A Patient's Own Story.

"On September 17 the general mobilisation was declared. When my friends and I heard about that, we all rejoiced, because we knew the purpose of the war—delivery of our brothers from the cruel tyrant. For a few days we got quite ready, and on September 24 we started with our regiment, decorated with flowers and the band playing exciting patriotic marches. We had to walk on foot five days and two days by train. When we entered the Turkish frontier we walked ninety kilometres and never heard shooting of guns. We approached Adrianople; during the night we made fortifications. The Turks were shooting on us, but in vain, because they were afraid to come out of their fortifications. On October 16 we faced the enormous Turkish army. The Turks, seeing that we were much less in number, thought of catching us all alive and taking us prisoners. But the Bulgarian soldiers are brave! Although the Turkish bullets were falling like hail and killing many of our brothers we were always proceeding forward. And then all at once our small army faced the great Turkish Division, and many of the Turks were killed by us. At the same time we got help. The fight went on without cease till the evening. The Turks were falling by hundreds. For six days we continued this kind of warfare, then the Servian soldiers came in our place while we started for Chataldja.

### The Battle at Chataldja.

"We walked for twelve days, night and day, almost without rest. On November 3 during the night we were at Chataldja. We just began to make our fortifications when we got the order to attack the Turks at once; they were quite far from us, and we walked nearly the whole night until we approached them. But unfortunately there was a river which separated us from the Turks. Several of us were half the body in the water; we had a hard struggle for three whole days and nights. Many of my friends were drowned in the water. In this same place our brave Captain —— was killed. Several soldiers were so unhappy at this that they put down their arms. Our situation was terrible. The enemy was attacking us from three different sides—night was coming on and we retired. At this time the Turks were doing all sorts of cruelties to our wounded soldiers; they cut off their ears, took out their eyes. They cut several organs from our good Lieutenant ——, whom they found wounded on the ground. At this same place I was wounded in the leg, and, thank God, I was taken from the field before the Turks had any time to torture me. I was taken to the next divisional hospital, which was so full that it was impossible for us to be taken in. So we had to spend seven days out of doors in the cold and rain. After the seventh day I was bandaged for the first time, and I felt much better after that. But I will never forget the dreary seven days during which I vomited constantly and ate almost nothing.

### Tribute to English Workers.

"The first hospital in which we were taken was Lozengrad, but it was so crowded that I longed to be moved from there. To my great joy we were sent to Sofia. For this purpose we had to go seven days in ox-carts and twenty-seven hours by train. So at present I am in the military school in Sofia, where I am very well looked after. I must say that I am most grateful to the capable and kind Dr. —— and his assistant doctors, and especially to the nice English sister Miss ——. And here in the military school I felt for the first time sure that I shall not die. And that is all that I can say."

### A Change, Indeed.

It seemed very different to an English hospital, and at first it was very trying always having a mob of twenty or thirty people crowding round to see what one was doing. The noise also was terrific—people tramping in and out all day long. The patients themselves were full of life; once over the worst, they were always singing, talking, and having fun together. Such picturesque beings they looked! For, on the whole, they are very fine-looking men. They wore a sort of white cotton pyjamas, and over these, half on, half off, the most gaily-coloured striped dressing-gowns of every shade under the sun. Once remark on their beauty, and the next time you would meet the same man with a flower over his ear or some other extra adornment! The men were such a help, too, with the nursing. They used to watch and take so much interest in everything, and would always come and fetch me if a patient wanted anything. There was a lot of massage to do, and this was a great time for learning Bulgarian; they used to love to teach us words, and I, in exchange, taught them English. Nothing pleased them more than to be able to say "Good morning, sister" or "A little better," when I said "How are you this morning?"

*(To be continued.)*

## Figures and Tables

**Figure f1:**
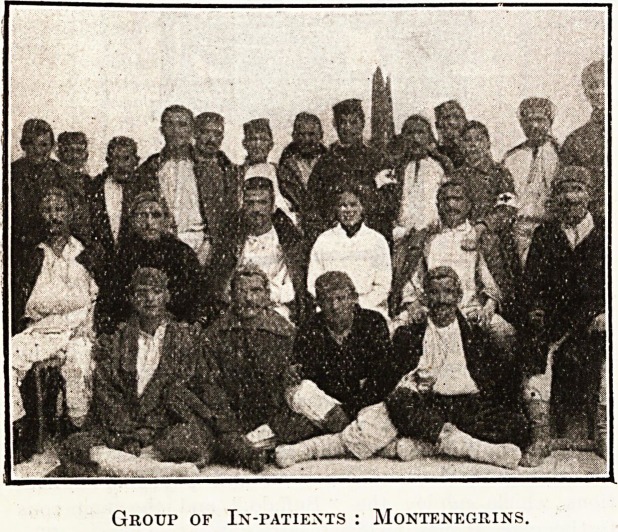


**Figure f2:**